# Sepsis Prediction at Emergency Department Triage Using Natural Language Processing: Retrospective Cohort Study

**DOI:** 10.2196/49784

**Published:** 2024-01-25

**Authors:** Felix Brann, Nicholas William Sterling, Stephanie O Frisch, Justin D Schrager

**Affiliations:** 1 Vital Software, Inc Claymont, DE United States; 2 Department of Emergency Medicine Emory University School of Medicine Atlanta, GA United States

**Keywords:** natural language processing, machine learning, sepsis, emergency department, triage

## Abstract

**Background:**

Despite its high lethality, sepsis can be difficult to detect on initial presentation to the emergency department (ED). Machine learning–based tools may provide avenues for earlier detection and lifesaving intervention.

**Objective:**

The study aimed to predict sepsis at the time of ED triage using natural language processing of nursing triage notes and available clinical data.

**Methods:**

We constructed a retrospective cohort of all 1,234,434 consecutive ED encounters in 2015-2021 from 4 separate clinically heterogeneous academically affiliated EDs. After exclusion criteria were applied, the final cohort included 1,059,386 adult ED encounters. The primary outcome criteria for sepsis were presumed severe infection and acute organ dysfunction. After vectorization and dimensional reduction of triage notes and clinical data available at triage, a decision tree–based ensemble (time-of-triage) model was trained to predict sepsis using the training subset (n=950,921). A separate (comprehensive) model was trained using these data and laboratory data, as it became available at 1-hour intervals, after triage. Model performances were evaluated using the test (n=108,465) subset.

**Results:**

Sepsis occurred in 35,318 encounters (incidence 3.45%). For sepsis prediction at the time of patient triage, using the primary definition, the area under the receiver operating characteristic curve (AUC) and macro *F*_1_-score for sepsis were 0.94 and 0.61, respectively. Sensitivity, specificity, and false positive rate were 0.87, 0.85, and 0.15, respectively. The time-of-triage model accurately predicted sepsis in 76% (1635/2150) of sepsis cases where sepsis screening was not initiated at triage and 97.5% (1630/1671) of cases where sepsis screening was initiated at triage. Positive and negative predictive values were 0.18 and 0.99, respectively. For sepsis prediction using laboratory data available each hour after ED arrival, the AUC peaked to 0.97 at 12 hours. Similar results were obtained when stratifying by hospital and when Centers for Disease Control and Prevention hospital toolkit for adult sepsis surveillance criteria were used to define sepsis. Among septic cases, sepsis was predicted in 36.1% (1375/3814), 49.9% (1902/3814), and 68.3% (2604/3814) of encounters, respectively, at 3, 2, and 1 hours prior to the first intravenous antibiotic order or where antibiotics where not ordered within the first 12 hours.

**Conclusions:**

Sepsis can accurately be predicted at ED presentation using nursing triage notes and clinical information available at the time of triage. This indicates that machine learning can facilitate timely and reliable alerting for intervention. Free-text data can improve the performance of predictive modeling at the time of triage and throughout the ED course.

## Introduction

### Background

Sepsis is a life-threatening condition caused by severe infection and dysregulated host response leading to acute organ dysfunction [1]. Affecting 32 million people and contributing to over 5 million deaths per year globally [2], sepsis is a leading cause of death in hospitalizations in the United States and worldwide [3,4]. Early antibiotics have been shown to improve survival [5], while each hour of delayed antibiotic administration has been associated with progressively increased mortality (7.6% increase per hour in septic shock) [6]. Patients who survive sepsis often have long-lasting health and social sequelae [7], and sepsis is ranked among the top 3 most costly conditions to treat in the hospital setting [8]. Accordingly, substantial efforts have been made to identify sepsis early in the hospital course [9]. To date, however, widely used clinical decision support tools that use rule-based methods for detecting sepsis have been limited by low sensitivity and specificity [10,11]. Such tools have been unable to earn clinician trust due to limited accuracy, false positives, and delayed alerts [12]. False positive alerts increase the cognitive load of providers and could expose patients to unnecessary antimicrobials. Moreover, current widely used electronic health record–based sepsis prediction tools have limited performance and often require several hours to elapse to achieve reasonable predictive use [12]. For example, a recent inpatient and intensive care unit (ICU)–based investigation of a commonly used sepsis alerting system showed that although existing systems can generate reasonably accurate sepsis alerts, the median time to notification was 7 hours and, even at that point, accuracy was limited [13]. Taken together, existing clinical decision support systems aimed at detecting sepsis do not provide sufficient accuracy or timeliness of sepsis prediction, resulting in lower adoption due to a lack of clinician trust.

### Machine Learning in Sepsis Prediction

Artificial intelligence (AI)–based tools may hold promise to increase the accuracy and timeliness of sepsis prediction, which may allow for earlier delivery of critical interventions such as lifesaving antibiotics. Many of the most promising sepsis predictive algorithms have been limited to use in ICU settings [14], where patients have rich laboratory and imaging data sets and frequent physiologic monitoring. In contrast, accurate prediction of sepsis at initial emergency department (ED) presentation has remained elusive. Until recently, there was a paucity of technology that could make use of the full set of available data, particularly free-text triage notes, at the time of initial ED presentation. A recent study showed that sepsis prediction at the time of triage can be significantly improved using natural language processing (NLP) of free-text data [15].

### ED Triage Assessment

When a patient presents to the ED, an initial triage assessment is usually performed by a triage nurse. The triage assessment includes a brief interview of the patient or those accompanying the patient to obtain a reason for presenting to the hospital ED. The content of this interview typically includes a very brief recounting of the patient’s past medical history, relevant medications, family history, and social risk factors. The triage nurse will typically also obtain vital signs (blood pressure, heart rate, temperature, respiratory rate, and oxygen saturation) and pain score. Finally, the triage nurse will assign a patient a triage acuity score. This process usually takes less than 10 minutes. The summation of this encounter is documented in real time, directly after the triage assessment, into the electronic medical record and includes a listing of the vital signs, triage acuity score, and a free-text nursing triage note.

The triage note is recorded into the electronic medical record, typically comprising 1-3 sentences regarding why the patient has presented to the ED and the nurse’s summative impression of this initial assessment. This note is used as a starting point for downstream assessments by providers in the ED. The information contained in the triage note is useful, as it often contains rich data that are difficult to quantify in tabular form. This information is widely used and valued by the clinical staff. However, in its unstructured format, it is not typically used in clinical decision support algorithms and is often unused for several hours until the full provider assessment. We hypothesized that nursing triage notes, combined with other data available at initial ED presentation, could be used to accurately predict sepsis at the time of triage.

### Goals of This Investigation

It was previously demonstrated that NLP of nursing triage notes at ED presentation could be used to predict hospital admission and ED resource use [16-18]. In this study, we aimed to demonstrate that an NLP-based model could be used to predict sepsis in adult patients based on the (1) health system sepsis committee and (2) Centers for Disease Control and Prevention (CDC) hospital toolkit for adult sepsis surveillance criteria [1].

## Methods

### Ethical Considerations

The research study protocol and procedures were reviewed and approved by the institutional review board (STUDY00000099).

### Study Design and Setting

A retrospective cohort was constructed using electronic health record data from all 1,234,434 consecutive ED encounters (487,296 unique patients) in 2015-2020 from 4 separate clinically heterogeneous academically affiliated EDs. Hospital A is a community hospital in an urban setting having a patient volume of approximately 65,000 ED visits per year. Hospital B is a community hospital in a suburban setting having a volume of approximately 26,000 visits per year. Hospital C is a quaternary care academic medical setting in a major metropolitan area having an ED patient volume of approximately 48,000 visits per year. Hospital D is a community hospital in a suburban setting having a volume of approximately 36,000 visits per year.

### Selection of Participants

Prior studies have suggested that overwhelming viral septicemia during the COVID-19 pandemic led to markedly increased false positive rates of sepsis screening tools [15]. These cases accounted for a substantial portion of ED visits during the initial months of 2020 [19] and led to a sharp decline in ED patient volume [20]. Accordingly, we excluded encounters (n=94,739) from February 1, 2020, to August 1, 2020, and patients who had a diagnostic code of COVID-19 or positive COVID-19 laboratory test. Patients of 18 years and younger of age were excluded from the study (n=27,238), as defining sepsis in these patients is controversial, and they are often lost to follow-up after they are transferred for admission to pediatric hospitals. Patients whose date of birth or age was not available were also excluded (n=23,434) to ensure that the remaining cohort comprised only adult patients. We subsequently excluded encounters with missing triage notes (n=29,637). The final cohort of interest included 1,059,386 unique clinical encounters.

### Sepsis Definition

The primary outcome of sepsis was defined as presumed severe infection and acute organ dysfunction, based on criteria described by the health system sepsis committee. To evaluate model performance against verified sepsis cases, the health system sepsis committee provided physician-reviewed sepsis labels for 7663 patients between June 1, 2019, and October 1, 2019. These cases were oversampled into the test data set. This definition of sepsis was projected onto the remaining data using clinical outcome variables. For sensitivity analyses of model performance, a secondary definition of sepsis was used, based on the US Centers for Medicare & Medicaid Services toolkit criteria [1]. Encounters were counted as sepsis, if they met criteria at any time during the ED course or hospital stay.

### Natural Language Processing

NLP techniques have been developed to extract meaning from unstructured free-text data. One such technique is document vectorization. Documents can be transformed into numerical vectors that represent the key information they contain, allowing them to be used by numerical machine learning (ML) techniques.

To generate document embeddings for the nursing triage notes, a distilled BERT (Bidirectional Encoder Representation From Transformers) model pretrained using an unsupervised masked language modeling objective was used as a base. Unlike models pretrained using a causal language modeling objective such as Generative Pre-Trained Transformer, which only consider preceding tokens, BERT considers tokens to the right and left of the masked word [21].

The use of large models such as BERT is constrained by the computational resources required for training and inference. DistilBERT [22] is a lighter and faster language model that offers fewer constraints on computational resources, having a depth of only 6 layers, rather than 12, and with token-type embeddings and pooler removed. DistilBERT is trained to replicate the behavior of BERT using “teacher-student” learning, where BERT is the “teacher” and DistilBERT is the “student.” This allows for knowledge distillation in the pretraining phase while retaining 97% of language understanding and being 60% faster.

The base DistilBERT model was fine-tuned using the free textual data from nursing triage notes with the objective of predicting sepsis. We evaluated several pretrained document vectorization models, selecting the optimal one by calculating fine-tuning performance on the training set. Nursing triage notes concatenated with Boolean clinical variables available at the time of triage (ie, high or low vital signs) were then passed through the fine-tuned DistilBERT model to produce document vectors representing the key information they contain. For the document vectors, we selected thresholds for the numeric values based on clinical knowledge and appended text based on the numeric values and those thresholds. Additionally, we developed manual mappings for known clinical abbreviations and converted them into the text. For example, “n/v/d” became “nausea, vomiting, and diarrhea.” The document vectors were then passed through a principal component analysis step to dimensionally reduce them from a length of 768 to 20 components.

### Model Training and Testing

For the time-of-triage model, the triage note vectors were combined with other clinical data, such as age, sex, and maximum and minimum vital signs. For the prediction of sepsis after laboratory data availability, a separate comprehensive model was constructed that included the aforementioned variables and additional laboratory data.

While many sepsis indicators have clear unidirectional associations with sepsis risk (ie, heart rate, hypotension, and lactic acid), others can be bidirectional (ie, high or low temperature or white blood cell [WBC] count). In addition, triage note vectors may potentially have complex relationships with sepsis. Accordingly, a decision tree–based technique was chosen for model training over more traditional techniques, such as logistic regression. The combined vectors from the training data set were used to train a decision tree–based ensemble learning model (XGBoost [Extreme Gradient Boosting]) [23] to predict the likelihood of sepsis. The XGBoost model was trained to predict sepsis using the training subset (n=950,921). Model performance was evaluated using the test (n=108,465) subset.

Optimal hyperparameters for the time-of-triage model were determined via grid search. The time-of-triage model was trained using a maximum tree depth of 6, minimum child weight of 15, minimum split loss of 15, learning rate of 0.05, subsample ratio of 0.6, L1 regularization of 0, and L2 regularization of 1. After Bayesian hyperparameter optimization, the comprehensive model was trained using a maximum tree depth of 6, minimum child weight of 13, minimum split loss of 18, learning rate of 0.015, subsample ratio of 0.63, L1 regularization of 0.27, and L2 regularization of 1.87. We accounted for class imbalance by scaling the positive weight parameter to the inverse of the class distribution. Epoch-level evaluation was used to measure model performance during training and identify failing training runs. Heat maps to indicate word and subword importance were generated using the integrated gradients method on the constructed model inputs [24]. Word importance here was calculated on words and subwords returned by the tokenization method.

For analysis of sensitivity, specificity, and false positive rate of the time-of-triage model, a target threshold of model prediction score was selected based on optimizing for a maximal false positive rate of 0.15. For the comprehensive model, we derived a classification threshold empirically, based on probability scores, and subsequently applied the threshold to target a maximum false positive rate of 0.1 at 12 hours after ED arrival. The thresholds were selected using model output scores from the training set and were applied to the test data set to evaluate clinical predictive performance metrics. The comprehensive model included known laboratory indicators of sepsis and end organ dysfunction, such as maximum and minimum WBC count, maximum lactic acid, minimum platelets, and maximum bilirubin and creatinine. Comprehensive model performance was evaluated using the test data set at every hour after ED arrival. Model performance was also evaluated at each hospital.

### Sepsis Prediction Prior to the First Intravenous Antibiotic Order

To estimate how an AI sepsis prediction tool might impact the ordering of antibiotics, we computed the percentage of sepsis encounters that triggered a positive prediction of sepsis prior to antibiotics being ordered or not having antibiotics ordered within the first 12 hours of the encounter. To perform this analysis, we used encounters from the test data set. A dual-model approach was used to emulate sepsis alerting at the time of triage and then subsequently during the ED encounter. Sepsis prediction time was defined as the earlier of either the time-of-triage model or comprehensive model generating a positive prediction of sepsis.

### Evaluation of Model Performances Among Clinically Undetected Sepsis Cases

To determine how the time-of-triage and comprehensive models may prevent missed sepsis, encounters with sepsis in the test data set were stratified by model prediction of sepsis- versus chart-based indicators of clinical sepsis suspicion. Predictive performance of the model was evaluated among patients who were septic and were or were not screened for sepsis at triage and defined as having either of the following order in less than 30 minutes after time of triage: (1) nursing-driven sepsis screening order set or (2) blood culture.

## Results

### Characteristics of the Study Patients

The total data set after exclusions consisted of 1,059,386 unique encounters from 487,296 patients. Sepsis occurred in 35,318 encounters (incidence 3.45%). Median time from arrival to first WBC count collection was 44.9 (IQR 26.2-79.3), 42.8 (IQR 25.6-73.3), and 44.8 (IQR 26.2-79.0) minutes across nonsepsis, sepsis, and all encounters, respectively. Demographic characteristics of the patients are available in Table 1. Gender, race, and temperature were missing in 5.6% (57,082/1,059,386), 13.2% (87,284/1,059,386), and 0.2% (2034/1,059,386) of encounters, respectively. Respiratory rate, heart rate, oxygen saturation, and blood pressure were missing in 0.1% of encounters. Selected examples of triage notes of encounters where patients were septic are included in Table S1 in Multimedia Appendix 1.

**Table 1:** Demographic and clinical characteristics of patients across encounters.

			Total		Hospital A		Hospital B		Hospital C		Hospital D
**Sepsis^a^, n (%)**			1,059,386 (100)		386,961 (36.5)		158,757 (15)		284,794 (26.9)		228,874 (21.6)
	Primary	35,318 (3.3)		9533 (2.5)		3978 (2.5)		12,775 (4.5)		9032 (3.9)	
	Secondary	31,542 (3)		9128 (2.4)		3541 (2.2)		12,688 (4.5)		6185 (2.7)	
**Age (years), mean (SD)**											
	18-24	80,384 (7.6)		35,421 (9.2)		11,466 (7.2)		23,309 (8.2)		10,188 (4.5)	
	25-44	344,034 (32.5)		147,085 (38.0)		47,283 (29.8)		91,106 (32.0)		58,560 (25.6)	
	45-64	327,584 (30.9)		123,225 (31.8)		53,226 (33.5)		87,113 (30.6)		64,020 (28.0)	
	65-74	141,943 (13.4)		44,840 (11.6)		19,709 (12.4)		41,425 (14.5)		35,969 (15.7)	
	≥75	165,441 (15.6)		36,390 (9.4)		27,073 (17.1)		41,841 (14.7)		60,137 (26.3)	
**Sex, n (%)**											
	Female	579,798 (57.8)		208,230 (56.8)		90,599 (60.4)		160,710 (59.6)		120,259 (55.6)	
	Male	422,506 (42.2)		158,321 (43.1)		59,447 (39.6)		108,611 (40.3)		96,127 (44.4)	
**Race, n (%)**											
	Black	552,432 (50.6)		301,619 (75.6)		35,366 (21.7)		150,454 (51.3)		64,993 (27.6)	
	White	380,084 (34.8)		53,427 (13.3)		92,713 (56.8)		104,290 (35.6)		129,654 (56.6)	
	Other	39,586 (36.3)		5205 (1.3)		15,827 (9.7)		10,125 (3.5)		8429 (3.6)	
	Unreported	87,284 (8.2)		26,710 (6.9)		14,851 (9.4)		19,925 (7.0)		25,798 (11.3)	
**Vital signs**											
	Temperature (°C), mean (SD)	36.8 (0.5)		36.8 (0.5)		36.8 (0.5)		36.7 (0.6)		36.8 (0.5)	
	Heart rate (beats per minute), mean (SD)	85.6 (18.8)		86.2 (18.1)		84.5 (18.7)		85.9 (19.1)		84.8 (19.7)	
	Systolic BP^b^ (mm Hg), mean (SD)	138.6 (26.7)		138.6 (26.9)		137.6 (24.4)		139.7 (28.9)		137.9 (24.9)	
	Diastolic BP (mm Hg), mean (SD)	80.0 (15.5)		80.8 (14.9)		80.2 (14.8)		80.5 (16.0)		77.8 (16.1)	
	SpO_2_^c^ (%), median (IQR)	98.0 (97-100)		98.0 (97-100)		98.0 (97-100)		98.0 (97-100)		99.0 (97-100)	
	Respiratory rate (breaths per minute), mean (SD)	18.0 (6.3)		18.2 (6.4)		18.0 (5.9)		18.1 (6.7)		17.8 (5.9)	
Time to first WBC^d^ count (minutes), median (IQR)			44.8 (26.5-80.3)		51.2 (27.3-85.0)		40.9 (20.8-62.8)		47.4 (32.4-90.3)		34.6 (23.1-73.0)

^a^Sepsis primary and secondary definitions based on the health system sepsis committee and Centers for Disease Control and Prevention hospital toolkit for adult sepsis surveillance criteria, respectively.

^b^BP: blood pressure.

^c^SpO_2_: oxygen saturation.

^d^WBC: white blood cell.

### Time-of-Triage and Comprehensive Model Performances

**Figure 1:**
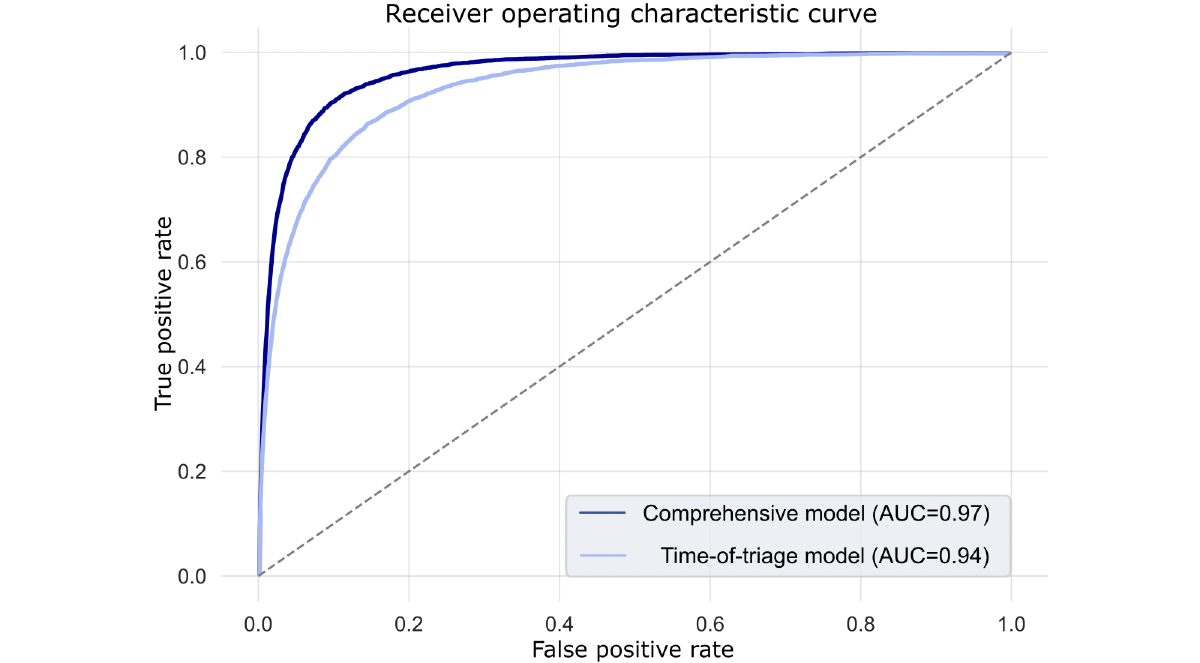
Receiver operating characteristic curve of sepsis prediction at the time of initial emergency department triage using free-text triage nursing notes and clinical data available at the time of triage. AUC: area under the receiver operating characteristic curve.

**Figure 2:**
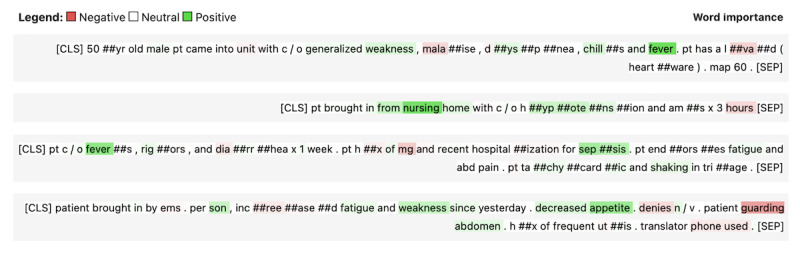
Heat maps applied to words and subwords of a sample of emergency department nursing triage notes to indicate relative contributions to sepsis prediction.

Using the test data set, the time-of-triage model using information available at initial triage for sepsis prediction (primary criteria) demonstrated an area under the receiver operating characteristic curve (AUC) and macro *F*_1_-score of 0.94 and 0.61, respectively (Figure 1). Sensitivity, specificity, and false positive rate were 0.87, 0.85, and 0.15, respectively. Positive and negative predictive values were 0.18 and 0.99, respectively. Sample model output is available in Figure 2, depicted as heat maps applied to words and subwords of ED nursing triage notes to indicate positive, neutral, or negative contributions to sepsis prediction.

**Figure 3:**
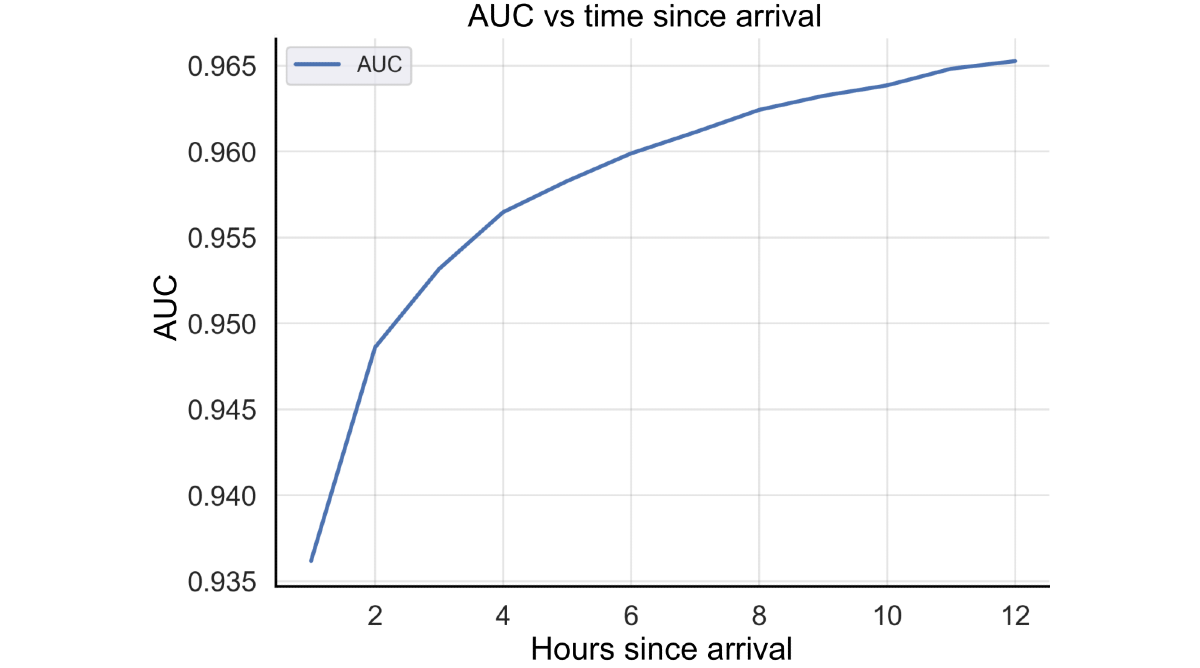
Sepsis predictive performance of the comprehensive model using a test data set, expressed as AUC, at each hour after emergency department arrival. AUC: area under the receiver operating characteristic curve.

Incorporating data available after initial ED workup, the comprehensive model predicted sepsis based on primary criteria with an initial AUC, sensitivity, and specificity of 0.94, 0.72, and 0.94 at 1 hour after ED arrival, respectively; increasing to an AUC, sensitivity, and specificity of 0.96, 0.87, and 0.91 after 5 hours, respectively; and increasing to AUC, sensitivity, and specificity of 0.97, 0.91, and 0.90 at 12 hours after arrival, respectively (Figure 3). Sensitivity, specificity, and false positive rate at 12 hours were 0.92, 0.89, and 0.11, respectively. Positive and negative predictive values at 12 hours were 0.25 and 0.99, respectively. Similar sepsis prediction results were obtained using the CDC hospital toolkit for adult sepsis surveillance criteria (Table 2) and when stratifying by hospital (Table S2 in Multimedia Appendix 1).

**Table 2:** Machine learning prediction of sepsis using data available at the time of emergency department (ED) triage (“time-of-triage” model) and all data available after ED workup (“comprehensive” model).

		Time-of-triage model	Comprehensive model
**Primary sepsis criteria**			
	AUC^a^	0.94	0.97
	Macro *F*_1_	0.61	0.67
	Sensitivity	0.87	0.91
	Specificity	0.85	0.90
	False positive rate	0.15	0.10
**CDC^b^ hospital toolkit for adult sepsis surveillance**			
	AUC	0.92	0.96
	Macro *F*_1_	0.57	0.64
	Sensitivity	0.86	0.91
	Specificity	0.83	0.89
	False positive rate	0.17	0.11

^a^AUC: area under the receiver operating characteristic curve.

^b^CDC: Centers for Disease Control and Prevention.

### Model Performances Among Clinically Undetected Sepsis Cases

Sepsis screening initiated at triage was defined as having chart-based indicators of sepsis screening ordered within 30 minutes of triage (see Methods section). Within the test data set, there were 3821 encounters having sepsis. Among these, 1671 (43.7%) encounters had sepsis screening initiated at triage. The time-of-triage model accurately predicted sepsis in 76% (1635/2150) of sepsis cases where sepsis screening was not initiated at triage and 97.5% (1630/1671) of cases where sepsis screening was initiated at triage.

### Model Performances Among Critical Sepsis Cases

Among patients in the test data set who had sepsis and were ultimately placed on vasopressors or were admitted to the ICU, the time-of-triage model predicted sepsis in 97.9% (329/336) and 91.6% (832/908) encounters, respectively. The comprehensive model predicted sepsis in 100% (336/336) and 95.7% (869/908) encounters, respectively.

### Sepsis Prediction Prior to the First Intravenous Antibiotic Order

We retrospectively evaluated the time of sepsis prediction in relation to the first intravenous antibiotic order using a dual-model approach (“time-of-triage” followed by “comprehensive” models). Among septic cases, sepsis was predicted in 36.1% (1375/3814), 49.9% (1902/3814), and 68.3% (2604/3814) of encounters at 3 hours, 2 hours, and 1 hour, respectively, prior to the first intravenous antibiotic order or where antibiotics were not ordered within the first 12 hours.

### Model Performance Using Only the First Encounter per Patient

To ensure that model performance was not confounded by past encounters, we performed a sensitivity analysis using only the first encounter per patient in the test data set (n=88,309), excluding subsequent encounters. The time-of-triage model predicted sepsis with an AUC, sensitivity, specificity, and false positive rate of 0.94, 0.85, 0.86, and 0.14, respectively. The comprehensive model predicted sepsis at 12 hours with an AUC, sensitivity, specificity, and false positive rate of 0.97, 0.92, 0.90, and 0.10, respectively.

### Analysis of Model Feature Importance

The importance of model features was analyzed by ranking the XGBoost feature importance scores from highest to lowest (Figure S1 in Multimedia Appendix 1). For both the time-of-triage (Figure S2 in Multimedia Appendix 1) and comprehensive (Figure S3 in Multimedia Appendix 1) models, the top features included elements of vital signs (ie, heart rate, temperature, blood pressure, and oxygen saturation) and triage note vectors. For the comprehensive model, the most important features additionally included laboratory metrics such as WBC count, creatinine, and lactic acid.

## Discussion

### Principal Findings

In this study, data from over 1 million patient encounters across 4 large metropolitan EDs were used to train an NLP-based ML model to detect sepsis at the time of patient presentation to the ED. We demonstrated that free-text nursing triage notes, combined with clinical variables at the time of triage, could be used to accurately predict the occurrence of sepsis at initial ED nursing triage. Moreover, we demonstrated that sepsis could be detected in 76% (1635/2150) of sepsis cases where sepsis screening was not initiated at triage. Finally, the results suggest that AI-based sepsis prediction in the ED may be able to significantly improve the time to antibiotics, which may offer opportunity for lifesaving intervention for patients. Notably, in addition to triage note vectors, the variables with the highest predictive importance were combinations of clinically relevant vital signs (time-of-triage model) and laboratory values, such as WBC count, creatinine, and lactic acid level (comprehensive model). These model characteristics, as well as the ability to map triage note word and subword relative contributions, indicate that the models may offer meaningfully explainable predictions to end users.

To our knowledge, this study is the largest to date to use NLP for sepsis prediction in the ED. We also demonstrated substantially improved accuracy compared to ML-based techniques in prior studies. The ability to incorporate triage notes into an ML model is advantageous for several reasons. First, natural language allows for a broad range of history and examination findings to be compressed into a short free-text note rather than innumerable variables in tabular form. Second, it allows experienced nurses to communicate an overall clinician impression that cannot always be captured by strictly quantitative inputs. In this study, free text from nursing triage notes was used to train a transformer model and was combined as input with other clinical data available at the time of initial triage, with the aim of predicting sepsis. Our findings demonstrate that NLP-based ML models can generate accurate predictions of sepsis at the time of triage and throughout an ED stay. Accordingly, the incorporation of free-text data into models that include data from clinical workups can produce a highly accurate prediction of sepsis.

### Importance of Accurate Sepsis Prediction Tools

Existing sepsis alerting systems experience a number of performance difficulties. One of the most widely implemented sepsis detection systems across health systems has been shown to have limited performance due to low sensitivity and precision (33% and 2.4%, respectively). Low predictive performance hinders the clinical use of such systems, despite their aim being to prompt the initiation of lifesaving care. Further impacting their use are high rates of false positive alerts [12]. Increased rates of false positive alerts lead to lower trust among clinicians, alert fatigue and dismissal, and lower adoption [25]. Recently, the incorporation of natural language such as free-text notes into model inputs has been shown to be promising for accurately detecting sepsis as early as during the ED triage process [15].

### Prior Studies

To our knowledge, this study is the largest to predict sepsis at the time of ED triage evaluation using NLP-based ML. Ivanov et al [15] reported high predictive performance for sepsis at ED triage with a smaller sample size in 2022. While both this study and Ivanov et al [15] present high sensitivity and specificity and remarkably increased performance compared to traditional screening tools for sepsis, there are important differences between the studies. Whereas Ivanov et al [15] included pediatric encounters, they were excluded in this study, since significantly ill patients of 18 years or younger of age are typically transferred to pediatric hospitals for admission and final diagnoses are unavailable. Accordingly, we excluded these encounters to avoid underestimation of sepsis in the pediatric population, which could have led to type I error with increased reliance on patient age as a predictive feature. A transformer model was also used for the NLP step, which can account for context and surrounding words.

Finally, our approach provides a method to present clinicians with understandable model decision explanations, including heat maps to indicate word importance and contribution to sepsis prediction. We present some examples of these heat maps here. It is important to note that the transformer architecture used in this study assigns meaning using full sentence context, capturing combined subword and interword relationships, from negation to more complex interactions. As such, these heat maps can be instructive but offer a heavily simplified view of how the algorithm uses triage notes. Additionally, the triage note vectorization is only a part of our complete sepsis algorithm, which also considers additional clinical data throughout the ED encounter.

### Limitations

There were several limitations in this study. First, physician-reviewed sepsis labels were only available for a subset of the data and had to be projected onto unlabeled encounters for training purposes using clinical signals. However, model performance was similar when evaluated on the secondary sepsis definition provided in the CDC hospital toolkit for adult sepsis surveillance. Second, the quality of the nursing triage notes is dependent on the clinical skill of the triage nurses, which could vary between EDs. Third, since the COVID-19 pandemic resulted in significant clinical and operational changes, it will be important to include such encounters in future prospective studies. Fourth, no pediatric patients were included, which would bias the model results toward an adult population. Fifth, in this study, it was not possible to detect whether patients were immunocompromised. This is an important subgroup of patients to assess in future studies of ML-based sepsis prediction. Sixth, it was not possible in this study to stratify by causal organism of sepsis, which could affect performance characteristics. Finally, as this study was an investigation of NLP using triage notes, we excluded encounters having missing triage notes.

### Conclusions

Using free-text and clinical data available at the time of initial ED triage from over 1 million patient encounters and across 4 hospital-based EDs, we demonstrated that NLP-based ML models are able to achieve high accuracy in predicting sepsis. The implication of these results is that AI-based clinical tools may substantially augment clinician abilities when clinical workup data are sparse, such as at the time of initial ED triage. Since sepsis mortality increases drastically with every passing hour and early clinical intervention is imperative to provide lifesaving treatment, AI-based tools using natural language data, such as free text available in nursing triage notes, may offer critical information to initiate treatment and prevent morbidity and mortality.

## Appendix

Multimedia Appendix 1 Examples of triage notes, subanalyses, and model explainability.

## Abbreviations

AI: artificial intelligence
AUC: area under the receiver operating characteristic curve
BERT: Bidirectional Encoder Representation From Transformers
CDC: Centers for Disease Control and Prevention
ED: emergency department
ICU: intensive care unit
ML: machine learning
NLP: natural language processing
WBC: white blood cell
XGBoost: Extreme Gradient Boosting
