# Supplementary material for: Sepsis Prediction at Emergency Department Triage Using Natural Language Processing: Retrospective Cohort Study
Source: JMIR AI. 2024 Jan 25;3:e49784. doi: 10.2196/49784 (PMC11041457; doi:10.2196/49784)
Supplement: Multimedia Appendix 1 [file ai_v3i1e49784_app1.docx]

**Multimedia Appendix 1**

**Table S1.** Selected examples of nursing triage notes from encounters with sepsis.

- *Reports shortness and generalized weakness last chemo on tuesday.*
- *Emergency medical services arrived for bradycardia weakness became v fib in emergency medical services shocked in emergency medical services and rhythm transitioned to asystole per emergency medical services lucus device started w cpr and epi given via io to left leg i gel intubate gi bleed per emergency medical services not currently on chemo.*
- *Emergency medical services states patient has history of endstage endometrial ca and states felt like was going to pass out patient hypotensive and vomiting on emergency medical services arrival patient recieved 200 cc fluids enorute.*
- *Patient reports vomitus altered mental status with loose stool x 1week.*
- *Patient brought in by emergency medical services per son increeased fatigue and weakness since yesterday decreased appetite denies n v patient guarding abdomen history of frequent urinary tract infections translator phone used.*
- *Right upper arm pain and swelling at central catheter line site recently hosp with sepsis here today from rehab facility noted to be hypotensive and tachycardic.*

**Table S2.** Machine learning prediction of sepsis, stratified by hospital ED.

|  | **Triage Time Model** | | | | |
| --- | --- | --- | --- | --- | --- |
|  |  |  |  |  |  |
|  | **Overall** | **Hospital A** | **Hospital B** | **Hospital C** | **Hospital D** |
| **AUC** | 0.94 | 0.94 | 0.94 | 0.92 | 0.93 |
| **Sensitivity** | 0.87 | 0.88 | 0.89 | 0.86 | 0.89 |
| **Specificity** | 0.85 | 0.85 | 0.86 | 0.82 | 0.83 |
| **FPR** | 0.15 | 0.15 | 0.14 | 0.18 | 0.17 |
|  | **Comprehensive Model** | | | | |
| **AUC** | 0.97 | 0.97 | 0.97 | 0.96 | 0.97 |
| **Sensitivity** | 0.92 | 0.91 | 0.91 | 0.91 | 0.92 |
| **Specificity** | 0.89 | 0.91 | 0.92 | 0.87 | 0.89 |
| **FPR** | 0.10 | 0.09 | 0.08 | 0.13 | 0.11 |

**Figure S1.** Ranked feature importance scores (top twelve) for time-of-triage and comprehensive models.

| **Time-of-Triage Model** |
| --- |
| **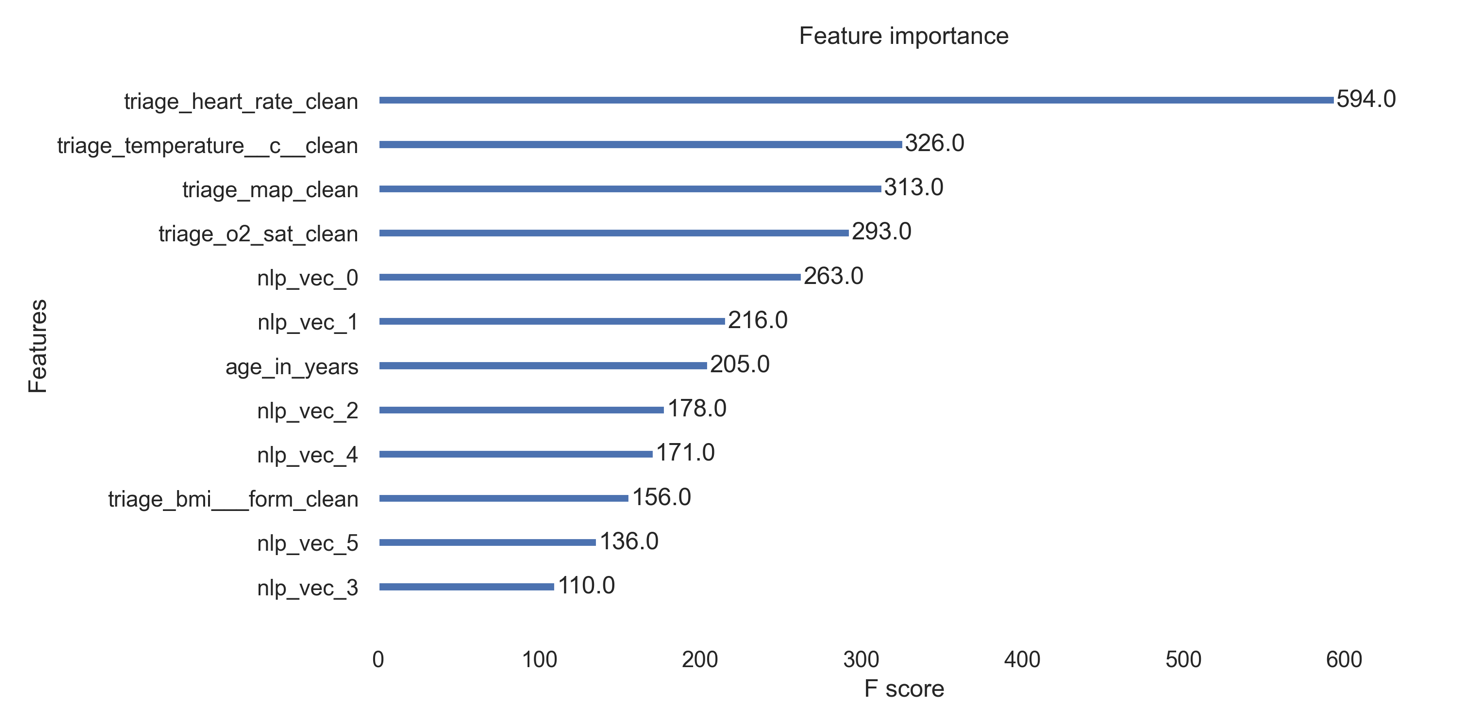** |
| **Comprehensive Model** |
| **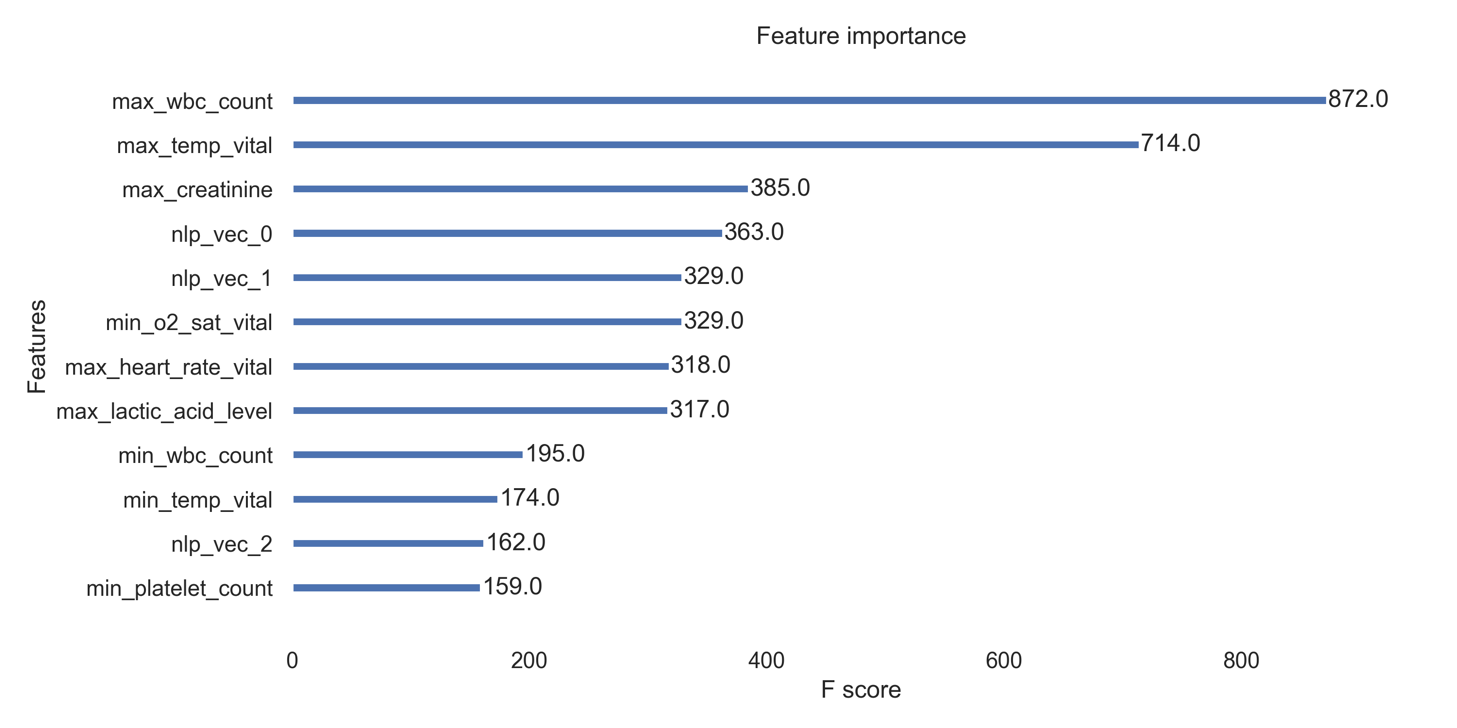** |

**Figure S2.** Partial dependence plots with individual conditional expectation for top ten most important features of time-of-triage model.
**
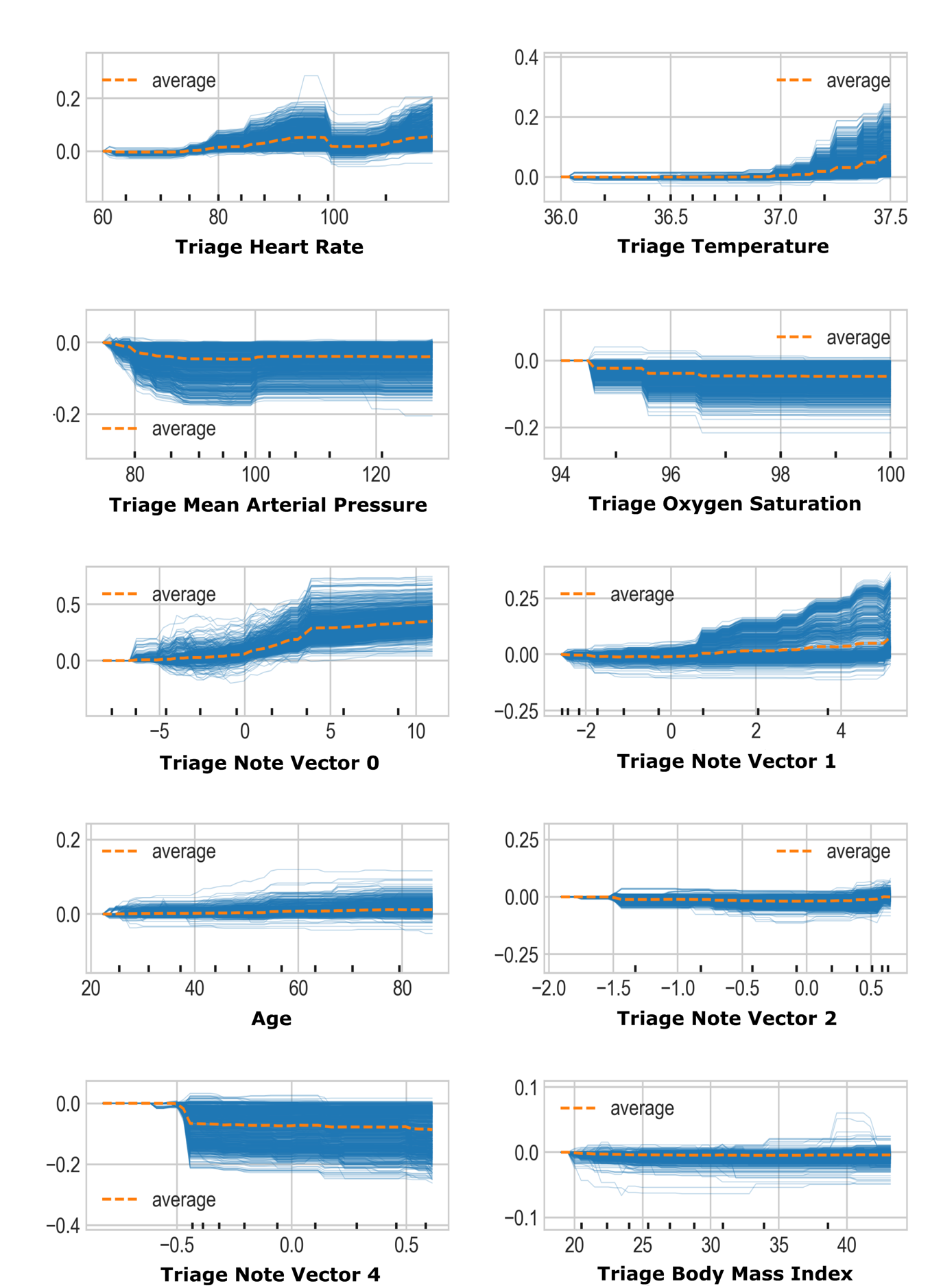
**

**Figure S3.** Partial dependence and individual conditional expectation plots for top ten most important features of comprehensive model.
**
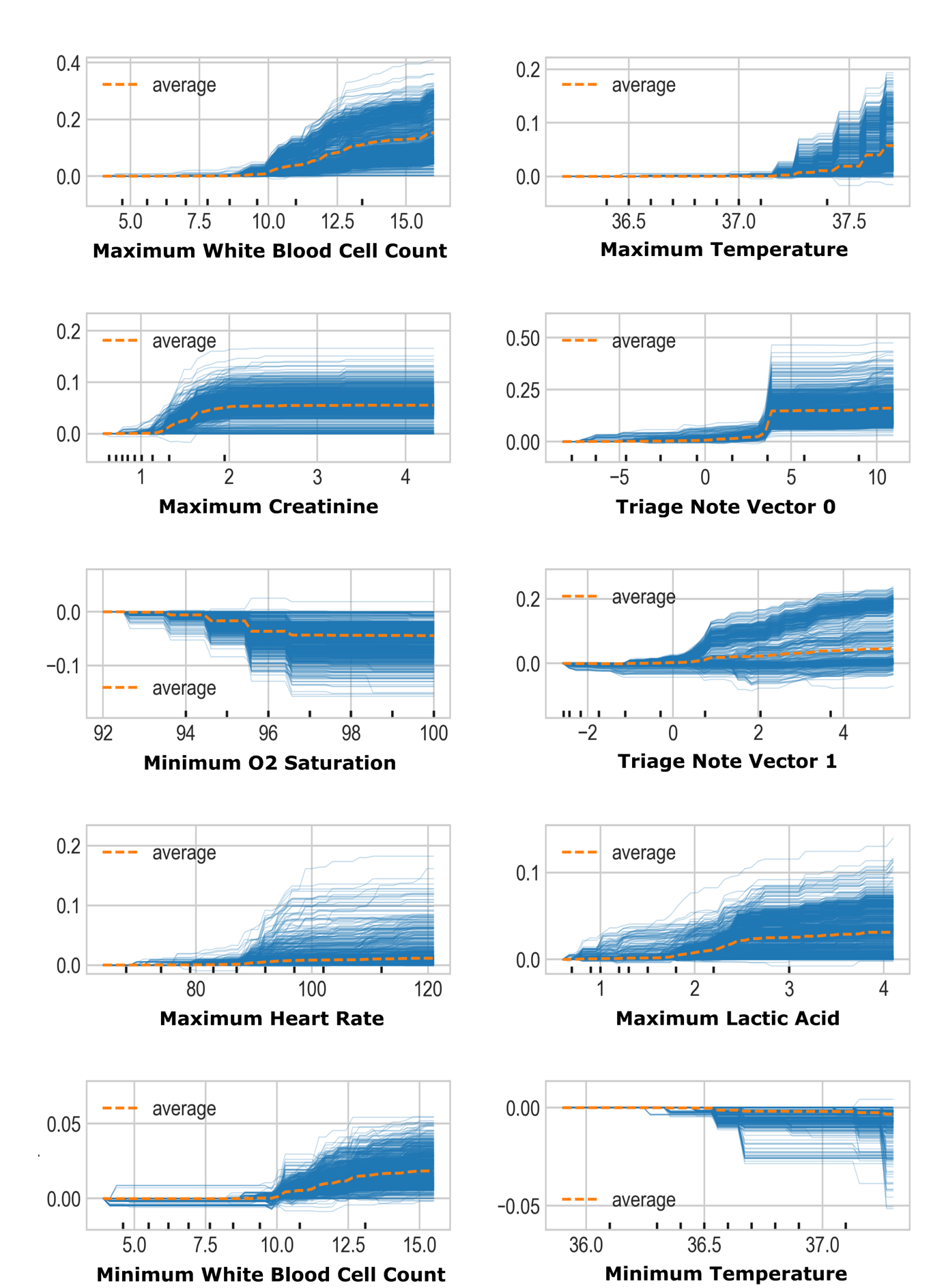
**
